# Structural Health Monitoring of a Reinforced Concrete Building during the Severe Typhoon Vicente in 2012

**DOI:** 10.1155/2013/509350

**Published:** 2013-10-24

**Authors:** Sin-Chi Kuok, Ka-Veng Yuen

**Affiliations:** Department of Civil and Environmental Engineering, University of Macau, Macau

## Abstract

The goal of this study is to investigate the structural performance of reinforced concrete building under the influence of severe typhoon. For this purpose, full-scale monitoring of a 22-story reinforced concrete building was conducted during the entire passage process of a severe typhoon “Vicente.” Vicente was the eighth tropical storm developed in the Western North Pacific Ocean and the South China Sea in 2012. Moreover, it was the strongest and most devastating typhoon that struck Macao since 1999. The overall duration of the typhoon affected period that lasted more than 70 hours and the typhoon eye region covered Macao for around one hour. The wind and structural response measurements were acquired throughout the entire typhoon affected period. The wind characteristics were analyzed using the measured wind data including the wind speed and wind direction time histories. Besides, the structural response measurements of the monitored building were utilized for modal identification using the Bayesian spectral density approach. Detailed analysis of the field data and the typhoon generated effects on the structural performance are discussed.

## 1. Introduction

Full-scale measurement is considered as a reliable approach for the evaluation of wind effects on structural systems. Although there has been significant progress on the techniques of wind tunnel tests and numerical modeling in recent decades, full-scale monitoring is irreplaceable for the study of some critical complicated phenomena, such as damping behavior, complex modes, and nonlinear structural behavior under severe excitation. Kijewski-Correa and Pirnia [1] chronicled the harvest of some representative full-scale monitoring projects conducted in various countries. It demonstrated the importance of full-scale monitoring for the study of the actual behavior of civil engineering infrastructures. Tropical cyclone generates violent aerodynamic excitation on infrastructures and causes considerable economic damage and human life loss to the affected coastal cities. Numerous research efforts have been devoted to analyzing the in-situ structural response measurements under such severe wind loading conditions. Xu and Zhan [2] and Li et al. [3] presented the field measurements for some landmarked buildings during the passage of severe typhoons. In [4], detailed analysis was presented for the typhoon effects on the wind load characteristics and structural responses of four super-tall buildings. In [5], the analytical and experimental modal analysis of the Guangzhou New TV Tower (renamed as the Canton Tower in 2010) was carried out and comparison of the structural performance was pursued under several typhoons. Other representative studies provided useful insights into wind-induced structural behavior under strong wind conditions [6–8].

The goal of this study is to investigate the structural performance of reinforced concrete buildings during the passage of severe typhoon since it is important for structural reliability evaluation [9–13]. This information will be useful for the improvement of structural design [14]. For this purpose, full-scale monitoring of a 22-story reinforced concrete building was conducted. One of the special features of this study is that this building has an asymmetric L-shape floor plan so the aerodynamic effects are more complicated than the ones studied in the literature. The monitoring period covered the entire passage process of the severe typhoon “Vicente.” It was the eighth tropical storm developed in the Western North Pacific Ocean and South China Sea in year 2012. By passing through Macao with the shortest core distance of only 40 km, Vicente is considered the strongest and most devastating typhoon for Macao since 1999. Since critical aerodynamic conditions were generated to the infrastructures in the city, it provided a valuable opportunity to investigate the structural behavior under harsh wind situation. In order to illustrate the wind conditions, the meteorological information, statistical properties of the wind measurements, and gust speed will be presented. Furthermore, the wind characteristic properties including the turbulence intensity and gust factor will be analyzed. 

On the other hand, the structural acceleration responses are analyzed and the Bayesian spectral density approach [15] is applied to identify the time-varying modal parameters. Bayesian inference provides a rigorous framework for parametric identification and has been applied to various engineering disciplines [8, 10–13, 16–18]. The Bayesian spectral density approach requires only the structural response measurement and it has been adopted for analyzing the structural response of the Canton tower [19]. One special feature of this frequency-domain approach is that it takes full advantage of Bayesian inference for the posterior uncertainty quantification of the modal estimation. As a result, the statistical uncertainties can be distinguished from the actual changes of the identified parameters. This is essential for reliable judgment about the structural integrity. In this paper, the time-varying modal parameters as well as the associated estimation uncertainties will be identified based on the structural response measurements of the entire monitoring period. 

This paper is organized as follows. In Section 2, the Bayesian spectral density approach for modal identification is briefly reviewed. In Section 3, the instrumentation on the monitored building is described. In Section 4, the aerodynamic properties of Vicente are presented. Finally, Section 5 presents the results and discussions about the typhoon effects on the structural performance of the monitored building.

## 2. Bayesian Spectral Density Approach for Modal Identification

Consider a linear dynamical system with *N*_*d*_ degrees of freedom:

(1) $$\begin{array}{c} M\ddot{x}+C\dot{x}+Kx=T_{0}F\left(t\right), \end{array}$$

where **M**, **C**, and **K** are the mass, damping, and stiffness matrix, respectively; and **T**_0_ is the force distributing matrix. The external excitation **F** can be modeled as zero-mean Gaussian white noise with spectral intensity matrix **S**_*F*_(*ω*) = **S**_*F*0_. The measurement **Y**_*N*_ = {**y**(*n*), *n* = 1,2,…, *N*} contains *N*_*o*_ channels of structural response, corrupted by the measurement noise **ε**:

(2) $$\begin{array}{c} y\left(n\right)=L_{0}q\left(n\right)+\varepsilon \left(n\right), \end{array}$$

where **y**(*n*) ∈ ℝ^*N*_*o*_^ is the measurement at the *n*th time step; **q**(*n*) ∈ ℝ^*N*_*d*_^ is the concerned structural response (e.g., acceleration) at the same time step; and **L**_0_ ∈ ℝ^*N*_*o*_×*N*_*d*_^ is the observation matrix comprised of zeros and ones. The measurement noise **ε** is modeled as zero-mean Gaussian independent and identical distributed (i.i.d.) process with covariance matrix Σ_**ε**_.

Use **α** to denote the uncertain modal parameter vector to be identified. It includes the structural modal parameters (i.e., modal frequencies, damping ratios, and mode shape components) of the contributing modes and the characteristic parameters of the spectral intensity of the excitation and the measurement noise. To identify the uncertain modal parameter vector **α**, a discrete estimator of the power spectral density matrix is utilized [15]:

(3) $$\begin{array}{c} S_{y,N}\left(\omega_{k}\right)=\frac{\Delta t}{2\pi N}\overset{N-1}{\underset{n,n^{\prime }=0}{\sum }}y\left(n\right){y\left(n^{\prime }\right)}^{T}\exp\left[-i\omega_{k}\left(n-n^{\prime }\right)\Delta t\right], \end{array}$$

where Δ*t* is the sampling time step; the superscript *T* denotes the transpose of a vector; Δ*ω* = 2*π*/(*N*Δ*t*) is the frequency precision in the discrete Fourier transform; and *ω*_*k*_ = *k*Δ*ω*, *k* = 0,1,…, INT(*N*/2). 

With *N*_*s*_ independent sets of discrete time histories **Y** = {**Y**_*N*_^(*s*)^, *s* = 1,2,…, *N*_*s*_}, the averaged spectral density matrix estimator can be obtained:

(4) $$\begin{array}{c} S_{y,N}^{\text{avg}}\left(\omega_{k}\right)=\frac{1}{N_{s}}\overset{N_{s}}{\underset{s=1}{\sum }}S_{y,N}^{\left(s\right)}\left(\omega_{k}\right), \end{array}$$

where **S**_*y*,*N*_^(*s*)^(*ω*_*k*_) can be calculated using (3) with the measurement **Y**_*N*_^(*s*)^. Given that *N*_*s*_ ≥ *N*_*o*_, the averaged spectral density matrix estimator follows the central complex *N*_*o*_-variate Wishart distribution with *N*_*s*_ degrees of freedom. With a properly selected frequency range *Ξ*, the averaged spectral density matrix estimators in **S**_*Ξ*_^avg^ = {**S**_*y*,*N*_^avg^(*ω*_*k*_), *ω*_*k*_ *ϵ* *Ξ*} are approximately independent [20].

Using the Bayes' theorem, the posterior probability density function (PDF) of **α** given **S**_*Ξ*_^avg^ is [16, 21]:

(5) $$\begin{array}{c} p\left(\alpha \;|\;S_{\Xi }^{\text{avg}}\right)=c_{1}p\left(\alpha \right)p\left(S_{\Xi }^{\text{avg}}\;|\;\alpha \right), \end{array}$$

where *c*_1_ is a normalizing constant. The prior PDF *p*(**α**) represents the prior information of the modal parameters in **α**. Throughout this study, it is taken as a noninformative prior distribution so it can be absorbed into the normalizing constant. The likelihood function *p*(**S**_*Ξ*_^avg^ | **α**) is given by the product of Wishart distributions [15]:

(6) p(SΞavg ∣ α) =c2∏ωk∈Ξ|E[Sy,N(ωk) ∣ α]|−Ns  ×exp⁡[−Nstr⁡({E[Sy,N(ωk) ∣ α]}−1Sy,Navg(ωk))],

where *c*_2_ is a constant that does not depend on the modal parameters; *E*[·] is the mathematical expectation; |·| and tr⁡(·) are the determinant and trace of a matrix, respectively. The optimal modal parameter vector $\hat{\alpha }$ can be determined by maximizing its posterior PDF. To enhance the computational condition, the optimal modal parameters can be obtained equivalently by minimizing the objective function defined as *J*(**α**) = −ln⁡*p*(**S**_*Ξ*_^avg^ | **α**). Consequently, the covariance matrix of the modal parameters is given by the inverse of the Hessian of *J*(**α**) evaluated at $\alpha =\hat{\alpha }$; that is, $\Sigma_{\alpha }={\left[H\left(\hat{\alpha }\right)\right]}^{-1}\equiv {\left[{\nabla J\left(\alpha \right)\nabla^{T}|}_{\alpha =\hat{\alpha }}\right]}^{-1}$ [22]. By employing the Bayesian spectral density approach with the measured structural acceleration response, the modal parameters and the associated estimation uncertainties can be determined. The associated uncertainty is important for the subsequent step of damage detection using modal parameters [23]. The Bayesian spectral density approach can be applied also for model updating of nonlinear dynamical systems [24].

## 3. Instrumentation of the East Asia Hall

**Figure 1:**
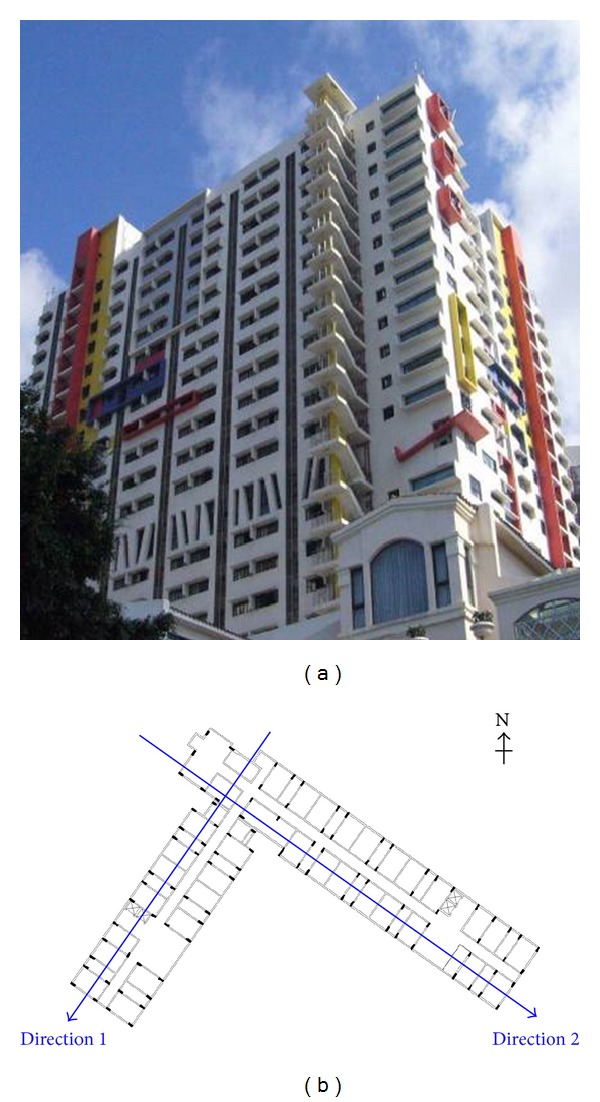
(a) Side-view and (b) typical floor plan of the East Asia Hall.

Full-scale measurement of a residential building, namely, the East Asia Hall, under the passage of typhoon Vicente is presented in this section. This building was inaugurated in 2005 for athletes lodging in the 4th East Asian Games hosted in Macao. Thereafter, it has been serving as a dormitory for the University of Macau.  Figure 1 shows the side view and a typical floor plan of the building. It is a 22-story reinforced concrete building of 64.70 m height, and its floor layer is in L-shape with span lengths 51.90 m and 61.75 m.

**Figure 2:**
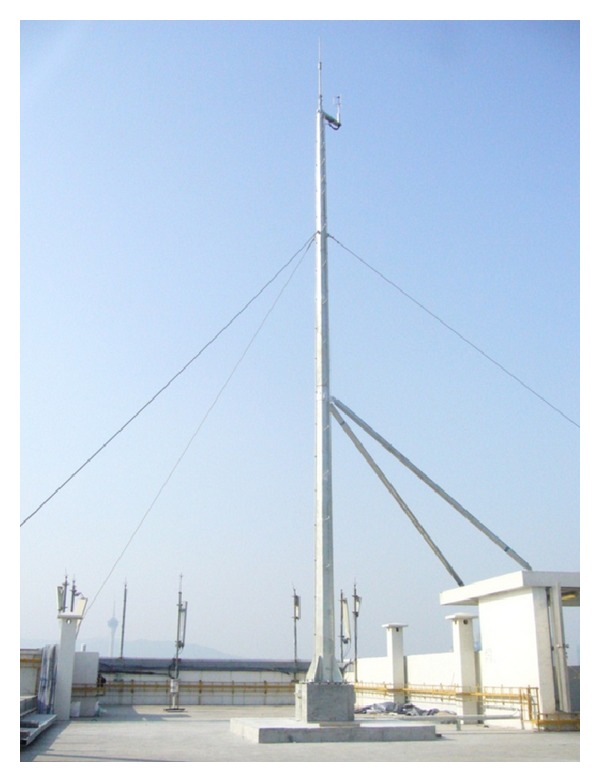
Photo of anemometer and its supporting mast.

A Gill-type ultrasonic anemometer was utilized to record the wind speed and wind direction time histories. It was mounted on a 10 m height mast at the top of the building (Figure 2). The resolution of the measured horizontal wind speed and wind direction was 0.01 m/s and 0.1°, respectively. Furthermore, the sampling frequency was 32 Hz. On the other hand, a biaxial state-of-the-art accelerometer was installed on the 18th floor (corresponding to 53.5 m height from the ground out of the total building height of 64.7 m) to capture the acceleration response of the building. It was placed at the junction of the two spans and the measured directions were indicated by the two perpendicular arrows in Figure 1(b). The accelerometer was operated under the standard exploration geophone spring-mass system with sensitivity of 50 V/g and sampling frequency of 200 Hz.

## 4. Typhoon Vicente

### 4.1. Meteorological Information

**Figure 3:**
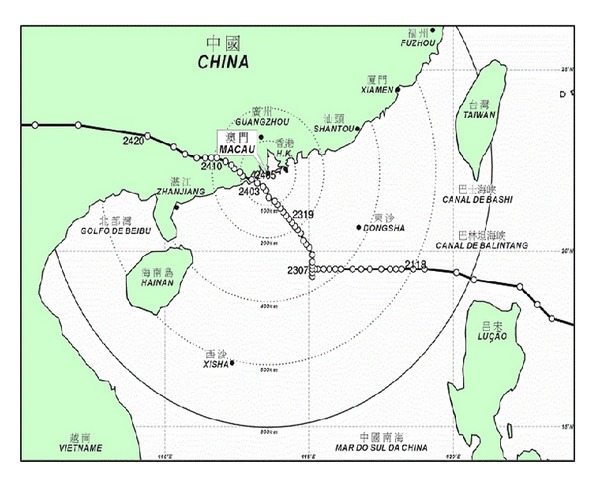
Track of typhoon Vicente (provided by the Meteorological and Geophysical Bureau (http://www.smg.gov.mo/www/e_index.php) and the time corresponding to GMT +08:00).

Typhoon Vicente developed as a tropical depression in the Northeast of Manila over the Western North Pacific Ocean on 20 July 2012. On the next day, it entered the South China Sea and the Standby Tropical Cyclone Signal Number 1 was announced in Macao by the Meteorological and Geophysical Bureau. On 22 July, Vicente stalled over the Northeastern region of the South China Sea and it was upgraded to a tropical storm. On 23 July, it began to edge towards the West of Pearl River Estuary of the South China coast. As the sustained wind speed became higher than 41 km/hr, the Signal Number 3 was released at 06:30. Vicente strengthened rapidly and attained to a severe typhoon. Along with the increasing wind speed, the Signal Number 8 was hoisted at 19:00 on the same day. At 02:15 on 24 July, the Signal Number 9 was issued due to the strength of the gale gust and storm force wind. Vicente passed through Macao from the South-Southwest with the shortest core distance of 40 km and its maximum near-center 10-minute sustained wind reached 150 km/hr. Afterwards, Vicente gradually moved away and made a landfall near the Taishan city in the Guangdong province. The signal number was subsequently downgraded to Number 8 and Number 3 at 05:00 and 09:30, respectively. The local wind was weakened during the dissipation of Vicente. Afterwards, all tropical cyclone signals were cancelled at 16:20. The typhoon track and the announced tropical cyclone signals of Vicente are summarized in Figure 3 and Table 1, respectively. From 18:00 on 21 July to 16:20 on 24 July, different tropical cyclone signals were hoisted due to the relevant typhoon generated wind situations. Full-scale monitoring was conducted for 70 hours and 20 minutes to cover the entire tropical cyclone signal hoisting period.

Vicente was the strongest and most devastating typhoon struck Macao since the severe typhoon York in 1999. It was also the only typhoon with Signal Number 9 or above from 2000 to 2013. It induced severe deterioration of the local weather. The daily precipitation of 143.2 mm was the highest record of July since 2001 and the pressure of 964.2 hPa was the lowest record since 1983. Moreover, the near eyewall region of Vicente covered Macao and the monitored building experienced the largest wind excitation in its entire history.

### 4.2. Wind Measurement

**Figure 4:**
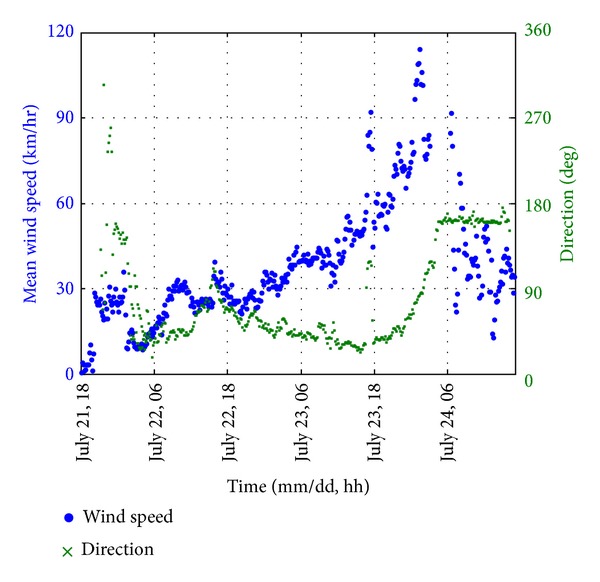
10-minute mean wind speed and wind direction.

The wind measurements, including the wind speed and wind direction obtained from the anemometer, are utilized for the wind analysis.  Figure 4 shows the 10-minute mean wind speed and the corresponding wind direction. The dots represent the mean wind speed while the crosses represent the wind direction. Herein, the wind direction is expressed in the azimuth, that is, counting clockwise from the true North. During the first 36.5 hours of the monitoring period, the Signal Number 1 was hoisted and the 10-minute mean wind speed was below 45 km/hr. Thereafter, the wind speed was increasing for the next twenty hours when Vicente was approaching. The dominated wind direction was Northeast. 

From 19:00 on 23 July to 09:30 on 24 July, the Signal Number 8 or above was hoisted. During this severe wind period, the 10-minute mean wind speed reached its maximum 114 km/hr and the wind direction changed gradually from Northeast to Southeast. After the Signal Number 8 was replaced by the Signal Number 3, the 10-minute mean wind speed became lower than 45 km/hr. Meanwhile, the wind direction remained in Southeast towards the end of the monitoring period and it merged to the background wind direction before the typhoon event.

**Figure 5:**
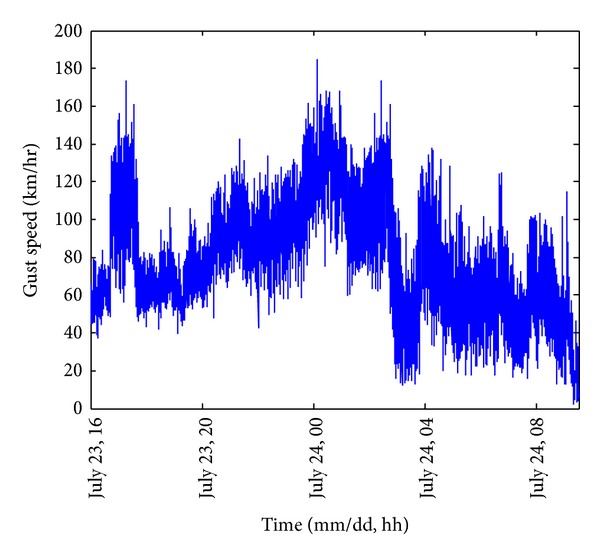
Gust speed of under severe wind load.

Figure 5 shows the gust speed under severe wind load. Gust speed is commonly utilized to illustrate the instant wind properties and it is defined as the highest wind speed over each 3-second interval. The maximum gust speed 185 km/hr occurred at the midnight of 24 July. On the other hand, the gust speed exhibited a manifest drop for an hour around 03:00 on 24 July. According to the typhoon track shown in Figure 1, Vicente arrived at the location of the shortest distance to Macao at that time. The measurements reveal that Macao entered the typhoon eye region, and hence the typhoon generated wind suddenly dropped from over 100 km/hr to the range from 20 km/hr to 60 km/hr. As Vicente was leaving, Macao no longer stayed inside the typhoon eye region and the gust speed recovered up to 140 km/hr. The recovery was not complete because Vicente started to land on the Guangdong coast at this stage.

### 4.3. Turbulence Intensity, Gust Factor, and Wind Spectra

Turbulence intensity and gust factor are widely adopted to represent the statistical features of the atmospheric turbulence and wind gustiness [25, 26]. The turbulence intensity *I*_*u*_ is defined as the ratio between the standard deviation of fluctuating wind *σ*_*u*_ and the mean wind speed $\overset{-}{U}$ calculated for each 10-minute interval [27]:

(7) $$\begin{array}{c} I_{u}=\frac{\sigma_{u}}{\overset{-}{U}}. \end{array}$$

On the other hand, the gust factor *G*_*u*_ is defined as the ratio between the maximum 3-second sustained wind speed $\max\left(\overset{-}{U_{3s}}\right)$ and the mean wind speed $\overset{-}{U}$ calculated for each 10-minute interval [27, 28]:

(8) $$\begin{array}{c} G_{u}=\frac{\max\left(\overset{-}{U_{3s}}\right)}{\overset{-}{U}}. \end{array}$$

**Figure 6:**
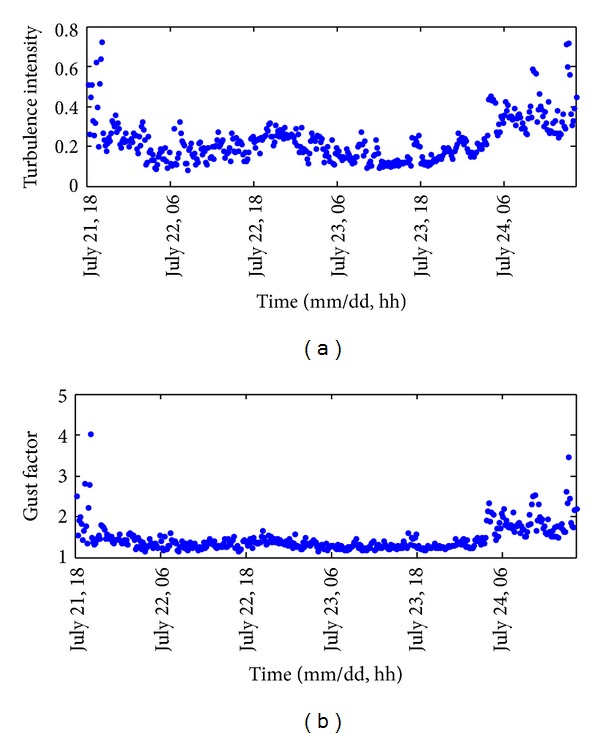
Time histories of (a) turbulence intensity and (b) gust factor.

**Figure 7:**
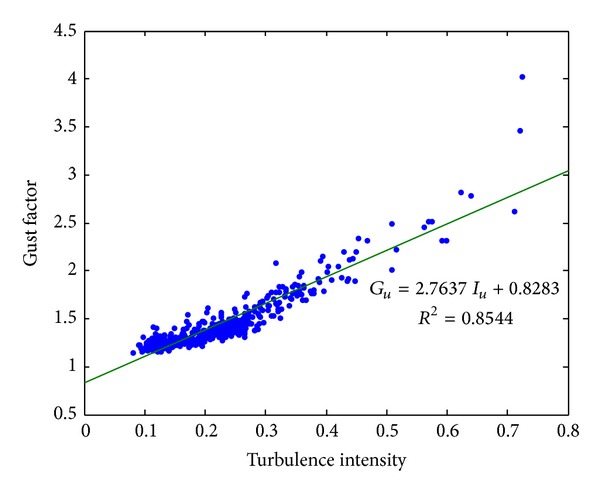
Gust factor versus turbulence intensity.

Figure 6 shows the time histories of the turbulence intensity and gust factor. Throughout the entire monitoring period, the turbulence intensity varied between 0.08 and 0.72 and the gust factor varied between 1.15 and 4.02. Both ratios were relatively more stable in the range of lower values associated with high wind speed. In contrast, their values at the beginning and ending stages (i.e., corresponding to relatively calm wind conditions) were larger and more fluctuating. Furthermore, the relationship between the gust factor and turbulence intensity is presented in Figure 7. The fluctuation of this severe typhoon is illustrated by the fact that there are a number of data points with gust factors over 2.0 and turbulence intensity over 0.5. Furthermore, it demonstrates clearly the strong positive correlation between these two quantities. This relationship can be well approximated as linear [27, 29] with the high coefficient of determination of *R* ^2^ = 0.8544. Moreover, it is observed that the data points on the top right corner, which is corresponding to the region of high gust factor and high turbulence intensity, are more scattering in comparison with the low-value region.

**Figure 8:**
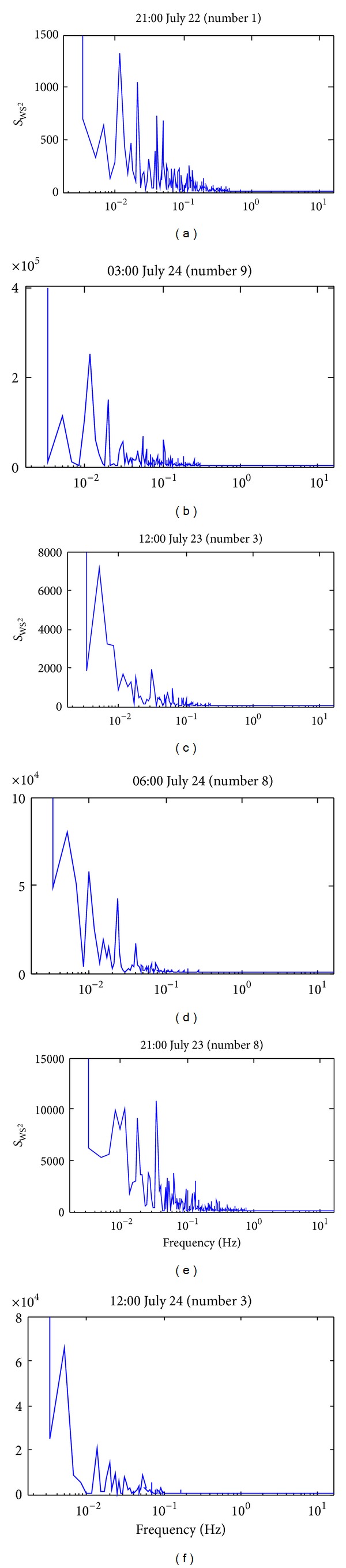
Wind speed square spectra under different tropical cyclone signals.

Figure 8 shows the spectra of wind speed square at different tropical cyclone signals. The wind speed square is used for demonstration because it is proportional to the induced wind pressure that affects the structural response. It can be seen that most of the frequency contents occurred below 0.1 Hz. Moreover, the frequency distribution varies for different tropical cyclone signals.

## 5. Structural Performance

### 5.1. Acceleration Response

**Figure 9:**
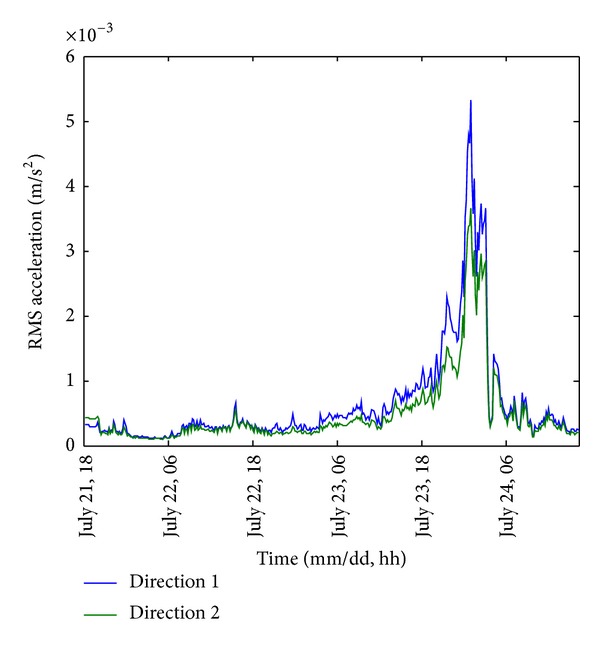
10-minute RMS structural acceleration response.

The acceleration responses of the building were measured in the two orthogonal directions shown in Figure 1(b). To illustrate the global trend of the structural response, the root-mean-square (RMS) acceleration is calculated for every 10-minute segment of the acceleration measurements and it is presented in Figure 9. These RMS accelerations lay within the interval [1.023 × 10^−4^, 5.322 × 10^−3^] m/s^2^ and [9.554 × 10^−5^, 3.654 × 10^−3^] m/s^2^ for direction 1 and 2, respectively. It was found that the RMS acceleration under severe wind load could be more than 10 times of the magnitude under Signal Number 1, inducing more than 100 times of wind load to the structure. Furthermore, it is observed that the acceleration responses in direction 1 were generally higher than that in direction 2. Due to the asymmetrical configuration of the building, the windward areas of the two spans are different. Moreover, the wind force contribution to the structural response depends highly on the attacking angle. It turns out that the difference is more significant when the strength of the wind is higher and the wind direction changes from Northeast to Southeast.

**Figure 10:**
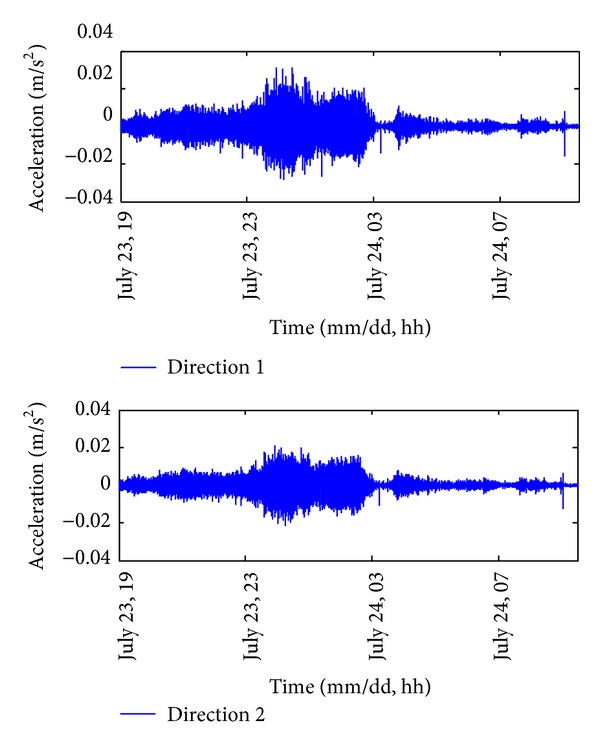
Acceleration response during the severe wind period.

In order to visualize the structural response during the severe wind period, the acceleration responses measured from 19:00 on 23 July to 09:30 on 24 July (i.e., the period when Signal Number 8 or above was hoisting) are shown in Figure 10. As expected, the amplitude of the acceleration response in direction 1 was generally higher than that in direction 2. Moreover, a notable reduction of the acceleration responses occurred for an hour around 03:00 on 24 July when the eye region of the typhoon entered Macao. Since the severe wind load was temporarily removed, the amplitude of the structural response was similar as that under Signal Number 1 only. It is worth noting that the observations are consistent with the conclusions drawn from Figure 5.

### 5.2. Modal Identification Results

The acceleration responses of the entire monitoring period are utilized for the modal identification. For each 10-minute segment of the acceleration responses, the Bayesian spectral density approach is applied to estimate the modal frequencies and damping ratios of the building and the spectral intensities of the wind excitation as well as their associated covariance matrices. Therefore, the variations of the identified modal parameters as well as the estimation uncertainties can be traced. 

**Figure 11:**
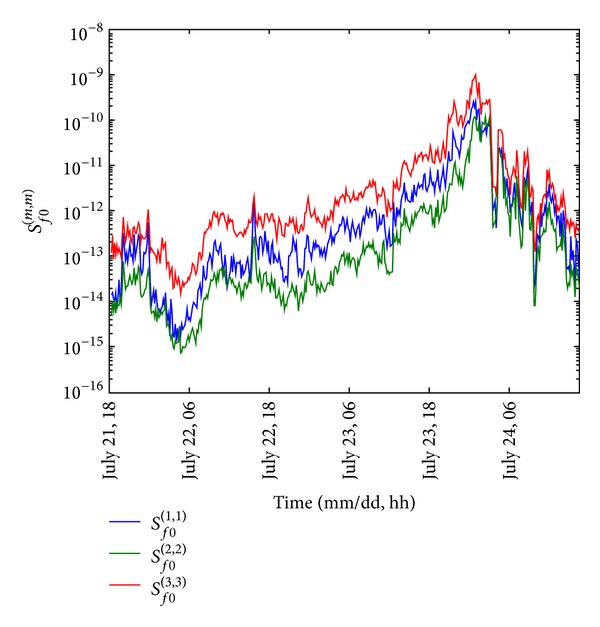
Time histories of the identified spectral intensities of the modal forces.

Spectral intensity of the modal force describes the excitation energy for a mode. The time histories of the identified spectral intensities of the modal forces for the first three modes are shown in Figure 11 with the semilogarithmic scale. It is found that the maximum values of the spectral intensities of the modal forces were thousand times higher than the corresponding values under Signal Number 1. Furthermore, the overall variations showed a similar trend as the 10-minute mean wind speed presented in Figure 4. For instance, the results continuously increased when Vicente was approaching Macao. A sudden drop of the excitation energy occurred when Macao was covered by the eye region of the typhoon. Then, the structural response decreased rapidly during the dissipation of the typhoon effect.

**Figure 12:**
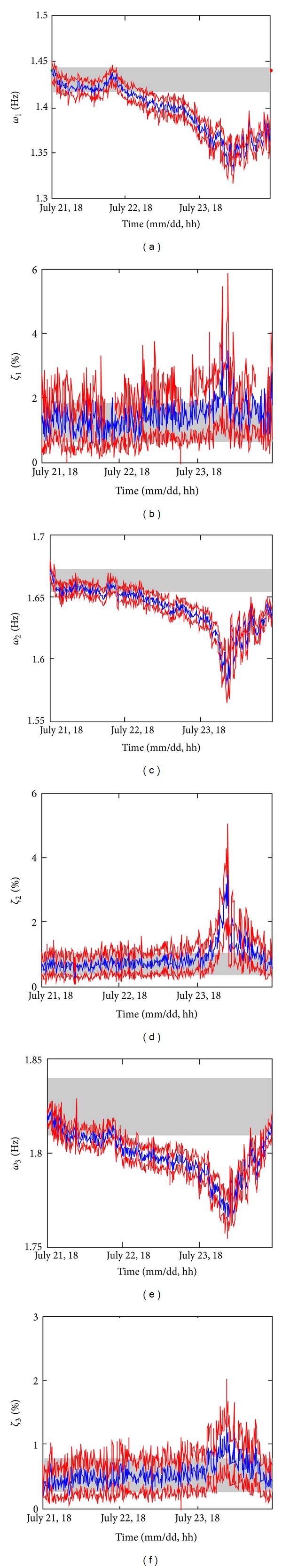
Time histories of the identified structural modal parameters.

Figure 12 shows the identified modal frequencies and damping ratios of the building with the associated estimation uncertainties. The identified modal frequencies are shown in the left column while the identified damping ratios are shown in the right column. The solid lines represent the identified values, and the dotted lines represent the plus and minus three standard derivations confidence intervals (i.e., ±3*σ* of the estimates) which yield a probability of 99.7%. Since the building may not behave linearly under the severe wind excitation, the identified structural modal parameters are referred to the corresponding quantities of the equivalent linear system.

Although the trends of the identified modal frequencies and damping ratios were opposite, all their peak values occurred approximately at the same time when the maximum values of the identified spectral intensities of the modal forces were achieved. Considerable reduction of the identified modal frequencies was observed during the severe wind period. The differences between the maximum and minimum of the three concerned modes were 8.35%, 5.90%, and 3.33%, respectively. Moreover, the standard deviations of the estimates were less than 0.5% for all the three modes. It turns out that the confidence intervals were sufficiently narrow compared with the variations of estimates throughout the monitoring period. This statistical evidence confirmed that, instead of the statistical uncertainty, there was notable reduction of the modal frequencies due to the severe wind load subjected to the building. 

On the other hand, the identified damping ratios of the three concerned modes were in the range of [0.50%, 3.73%], [0.19%, 3.59%], and [0.17%, 1.43%], respectively. The associated maximum standard derivations were 0.80%, 0.48%, and 0.23%, respectively. The fluctuation of the estimates and the estimation uncertainties were significantly larger than those for the modal frequencies. Nevertheless, it is sufficient to conclude that the damping ratios were higher during the severe wind period. 

### 5.3. Investigation of Permanent Effect

Next, the identified structural modal parameters at the beginning and the end of the monitoring period are compared. From Figure 12, it is realized that some of the modal frequencies were not fully recovered even after the typhoon generated wind excitation was dissipated. The largest difference is found to be 4.69% for the identified modal frequency of the first mode. The two major sources of this phenomenon are the dramatic change of the environmental conditions and the nonlinear behavior of the structure. Due to the atmospheric mechanism of tropical cyclones, the scorching weather at the early stage of the typhoon event changes to the cool and showering condition afterwards. Hence, there were notable differences in the corresponding environmental conditions of the temperature and relative humidity. For instance, the daily average temperature and relative humidity on 21 July and 24 July were (30.8°C, 74%) and (26.0°C, 92%), respectively. Previous studies revealed that the structural modal frequencies depend on the environmental conditions [30, 31]. Therefore, direct comparison between the modal frequencies should be conducted under same environmental conditions. Taking this into account, the identified structural modal parameters obtained on 7 August 2012, which was two weeks after the typhoon event, is utilized for comparison. The environmental conditions of this day returned to subtropical climate with hot and humid summer weather and the environmental conditions were similar to the conditions at the beginning of the typhoon event. In particular, the daily average temperature and relative humidity were 29.5°C and 79%, respectively. Therefore, indication of possible permanent effects on the building can be achieved by comparing the modal frequencies of this day with the beginning of the typhoon. Again, the modal frequencies and damping ratios were identified for every 10-minute response measurement on 7 August and the shaded rectangles in Figure 12 enclose the ranges for each modal parameter. It is found that they covered the ranges of the early stage of the typhoon event so it indicates that all the modal frequencies and damping ratios recovered to their original levels when similar environmental conditions are encountered after the typhoon. In other words, it confirms that no permanent effect was induced by Vicente to the building. 

**Figure 13:**
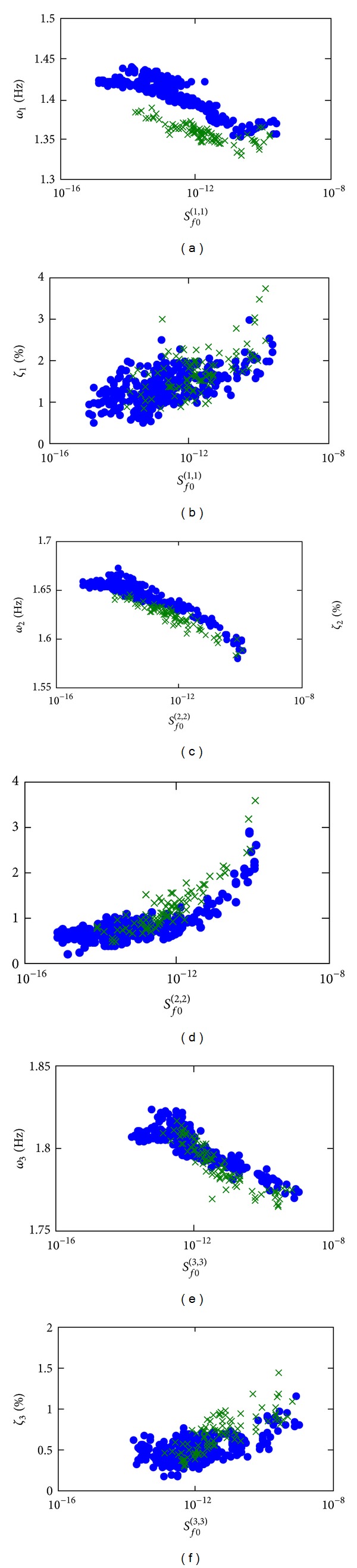
Identified structural modal parameters versus the identified spectral intensity of the modal force.

In order to demonstrate the hysteretic behavior of the structure, the relationships between the identified structural modal parameters and the identified spectral intensities of the corresponding modal forces are presented in Figure 13. The data points under the loading and releasing process are represented by the dots and crosses, respectively. Herein, the loading process is referred to the stage with increasing 10-minute RMS structural response while the releasing process is referred to the latter stage with decreasing 10-minute RMS structural response. The left column shows the identified modal frequencies versus the identified spectral intensities of the corresponding modal forces. For all three modes, the identified modal frequencies had a decreasing trend with the spectral intensities. This indicates certain nonlinear behavior of the building. Reduction of the equivalent linear stiffness indicates the nonlinear behavior of this reinforced concrete building under severe wind load. As a result, downward trends can be observed in all these figures in the left column. Moreover, it is observed that the modal frequencies were generally lower in the releasing process when the spectral intensities were high. It is noted that the ambient temperature and relative humidity were stable during the severe wind period, so the variation of the structural properties due to the environmental influences were negligible at this stage. One possible explanation is that the hysteretic behavior of the structure for the loading and releasing process was different and this is commonly observed for reinforced concrete structures [32, 33]. When the spectral intensities were decreasing, the modal frequencies in the releasing process were lower than the corresponding values with the same level of excitation energy in the loading process.

The identified damping ratios versus the identified spectral intensities of the corresponding modal forces are shown on the right column in Figure 13. For all the three modes, increasing trends were observed as the excitation energy increased. This observation reconfirms the nonlinear hysteretic behavior of the building. As a result, energy was dissipated more efficiently through the hysteretic loops so the equivalent damping ratios were significantly increased under severe wind condition. Moreover, it is found that the identified damping ratios of the releasing process were slightly larger than those of the loading process when the identified spectral intensities of the modal forces were high. The temporary increase of the damping ratios vanished when the excitation energy returned to the calm wind conditions.

**Figure 14:**
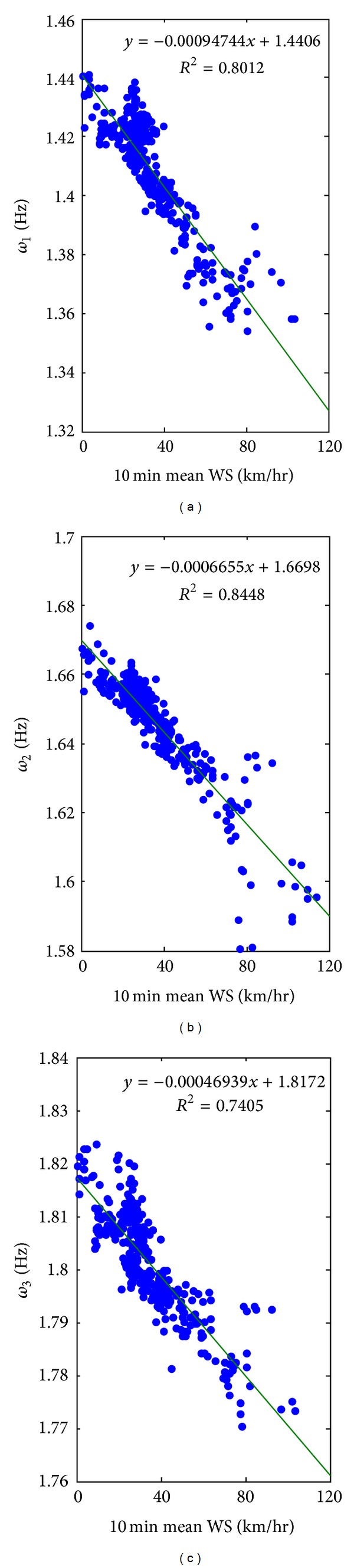
Identified modal frequencies versus 10-minute mean wind speed.

Figure 14 shows the identified modal frequencies versus the 10-minute mean wind speed during the loading process. For all the three concerned modes, decreasing trends are observed. It is found that the data points are distributed more closely along the trend lines for the low wind speed region. When the wind speed was higher than 60 km/hr, the data points became more scattering. It turns out that the coefficients of determination (*R* ^2^) of the three modes are 0.8012, 0.8448, and 0.7405, respectively, and they provide strong evidence of the nonlinear behavior of the building during strong wind load.

## 6. Conclusion

In this study, the typhoon effects on the structural performance of a reinforced concrete building were investigated. Wind and structural response measurements were acquired throughout the passage of the severe typhoon Vicente. It was the strongest typhoon for Macao since 1999. During the monitoring period, Macao was covered by the typhoon eye region for around an hour. The measured data were utilized to evaluate the wind characteristics as well as the structural properties throughout the monitoring period. The magnitude of the acceleration response under Signal Number 9 could be more than 10 times higher than that under Signal Number 1. When the monitored building was inside the typhoon eye region of Vicente, both the wind speed and structural response measurements were temporarily decreased for a significant amount. Furthermore, the structural responses were utilized for modal identification using the Bayesian spectral density approach. As the spectral intensities of the modal forces increased, the modal frequencies decreased but the damping ratios increased. During the severe wind excitation, nonlinear hysteretic behavior of the structure was observed but no permanent effect was induced. 

## Figures and Tables

**Table 1:** Announced tropical cyclone signals of Vicente by the Macao Meteorological and Geophysical Bureau.

Tropical cyclone signal	Sustained wind speed (km/hr)	Gust speed (km/hr)	Hosting time (hr:minute mm/dd, GMT +08:00)	Duration
1	<41	—	18:00 07/21	36 hr 30 min
3	[41, 62]	110	06:30 07/23	12 hr 30 min
8	[63, 117]	180	19:00 07/23	7 hr 15 min
9	<118	—	02:15 07/24	2 hr 45 min
8	[63, 117]	180	05:00 07/24	4 hr 30 min
3	[41, 62]	110	09:30 07/24	6 hr 50 min
0	<41	—	16:20 07/24	—
